# Supplementary material for: Neural Correlates of Dynamic Predictions and Prediction Errors in Response to Unexpected Silence and Sound
Source: Eur J Neurosci. 2026 Feb 25;63(4):e70422. doi: 10.1111/ejn.70422 (PMC12935523; doi:10.1111/ejn.70422)
Supplement: Supplementary file 1 — Table A1: Descriptive statistics for included trials per trial type. M = mean, SD = standard deviation. [file EJN-63-0-s001.docx]

# Supplementary

| Type of trial | Median | *M* | *SD* | Range |
| --- | --- | --- | --- | --- |
| predicted sound trials | 170 | 169 | 36.9 | 92 - 342 |
| predicted omission trials | 166 | 168 | 38.4 | 84 - 328 |
| mispredicted sound trials | 74 | 74 | 13.9 | 40 - 102 |
| mispredicted omission trials | 80 | 76 | 11.9 | 40 - 89 |
| unpredictable sound trials | 75 | 72 | 13.9 | 32 - 92 |
| unpredictable omission trials | 77 | 72 | 15.4 | 27 - 88 |

**Table A1:** Descriptive statistics for included trials per trial type. M = mean, SD = standard deviation
